# Comparing the performance of selected variant callers using synthetic data and genome segmentation

**DOI:** 10.1186/s12859-018-2440-7

**Published:** 2018-11-19

**Authors:** Xiaopeng Bian, Bin Zhu, Mingyi Wang, Ying Hu, Qingrong Chen, Cu Nguyen, Belynda Hicks, Daoud Meerzaman

**Affiliations:** 1 Center for Biomedical Informatics and Information Technology, National Cancer Institute, Rockville, MD 20850 USA; 2 Cancer Genomics Research Laboratory(CGR), Division of Cancer Epidemiology and Genetics, Frederick National Laboratory for Cancer Research sponsored by the National Cancer Institute, 8717 Grovemont Circle, Gaithersburg, MD 20877 USA

## Abstract

**Background:**

High-throughput sequencing has rapidly become an essential part of precision cancer medicine. But validating results obtained from analyzing and interpreting genomic data remains a rate-limiting factor. The gold standard, of course, remains manual validation by expert panels, which is not without its weaknesses, namely high costs in both funding and time as well as the necessarily selective nature of manual validation. But it may be possible to develop more economical, complementary means of validation. In this study we employed four synthetic data sets (variants with known mutations spiked into specific genomic locations) of increasing complexity to assess the sensitivity, specificity, and balanced accuracy of five open-source variant callers: FreeBayes v1.0, VarDict v11.5.1, MuTect v1.1.7, MuTect2, and MuSE v1.0rc. FreeBayes, VarDict, and MuTect were run in bcbio-next gen, and the results were integrated into a single Ensemble call set. The known mutations provided a level of “ground truth” against which we evaluated variant-caller performance. We further facilitated the comparison and evaluation by segmenting the whole genome into 10,000,000 base-pair fragments which yielded 316 segments.

**Results:**

Differences among the numbers of true positives were small among the callers, but the numbers of false positives varied much more when the tools were used to analyze sets one through three. Both FreeBayes and VarDict produced strikingly more false positives than did the others, although VarDict, somewhat paradoxically also produced the highest number of true positives. The Ensemble approach yielded results characterized by higher specificity and balanced accuracy and fewer false positives than did any of the five tools used alone. Sensitivity and specificity, however, declined for all five callers as the complexity of the data sets increased, but we did not uncover anything more than limited, weak correlations between caller performance and certain DNA structural features: gene density and guanine-cytosine content. Altogether, MuTect2 performed the best among the callers tested, followed by MuSE and MuTect.

**Conclusions:**

Spiking data sets with specific mutations –single-nucleotide variations (SNVs), single-nucleotide polymorphisms (SNPs), or structural variations (SVs) in this study—at known locations in the genome provides an effective and economical way to compare data analyzed by variant callers with ground truth. The method constitutes a viable alternative to the prolonged, expensive, and noncomprehensive assessment by expert panels. It should be further developed and refined, as should other comparatively “lightweight” methods of assessing accuracy. Given that the scientific community has not yet established gold standards for validating NGS-related technologies such as variant callers, developing multiple alternative means for verifying variant-caller accuracy will eventually lead to the establishment of higher-quality standards than could be achieved by prematurely limiting the range of innovative methods explored by members of the community.

## Background

Next-generation sequencing (NGS) has begun to transform molecularly based precision cancer medicine into a clinical reality [1, 2]. Sequencing results already influence how physicians diagnose patients, perform prognostic evaluations, decide among therapeutic choices, and provide guidance on prevention strategies to cancer patients or people at risk of cancer development or recurrence. It is essential that such clinical decisions be based upon the valid identification of variations in the genetic makeup of individual patients [3].

A relatively large number of open-source informatics tools for identifying, or calling, genomic variants is now available to basic scientists and other researchers. Most often, these tools feature different algorithms, filtering strategies, and recommendations for use, which together lead to different outputs [4, 5]. However, the literature offers limited guidance for researchers attempting to efficiently select those tools which offer a level of certainty high enough to approach the standards of good clinical practice.

Many studies analyzing, comparing, and evaluating variant-caller performance have been reported [4, 6–15]. While analysis and comparison of selected variant callers has become more expeditious in some respects, determining validity, the correspondence between outputs and biological reality, has generally remained laborious. Validation, usually through Sanger sequencing, remains notably expensive and time-consuming; it also demands an array of laboratory and clinical resources. To generate large sets of “real” data, tumor samples must be taken from patients, then processed and sequenced. Verifying sequences requires expertise and time, usually in the form of panels of experts who manually compare variant-caller outputs with real sequencing data in the laboratory. Moreover, manual evaluations are necessarily limited to a small fraction of all detected variants [6, 9, 12]. As a result, assessing validity too often becomes a bottleneck, slowing assessment of variant callers and other high-throughput research technologies, as well as the translation of technologies such as these into clinical settings.

However, it may be possible to develop additional methods of validation that would be more practically suited to assessing not only the overall performance but also more precisely the specificity and sensitivity of variant callers. In this study, by using data sets with known mutations at specific sites, we explore the effectiveness of using synthetic data sets to provide a level of ground truth against which results can be validated. Our final results were obtained from analyzing, comparing, and evaluating the performance of five widely used open-source variant callers that identify single-nucleotide variants (SNVs).

The data used comprised three well-known synthetic sets originally created for the 2014 International Cancer Genome Consortium (ICG-C)-TCGA Dialogue for Reverse Engineering Assessments and Methods (DREAM) Somatic Mutation Calling Challenge plus a more complex set created more recently following the same method [16]. The sets were created using a binary alignment map (BAM) file derived from a tumor cell line as the foundation. Mutated sequences were inserted at specific genomic locations, thus providing ground-truth indicators that can be rapidly verified.

Finally, by splitting the genome into 10 million nucleotide segments, we were also able to begin exploring the questions of (1) whether different callers might perform differently on different parts of the genome, specifically whether gene density (a measure of the number of genes per the number of base pairs) or guanine-cytosine (G-C) content (a marker of intergenic homopolymeric sequences) might affect results; and (2) whether tool performance might vary in the same or in different locations.

## Methods

### Data sets

The four data sets can be downloaded from the DREAM challenge site [16]. To create these data sets, the DREAM team cited above developed BAMSurgeon, an open-source tool for accurate tumor-genome simulation that adds synthetic mutations directly to existing reads in the BAM format [4]. It can spike in mutations at selected positions at any allelic fraction or simulate multiple subclones and instances of sample contamination. The team used a deeply sequenced (> 60-80x coverage) BAM file from a tumor-cell line. Two equally sized subsets were generated from the BAM file, one of which was left intact and a second which was spiked in with known mutations at specific genomic locations. The new BAM files thus generated are designated as “tumor”; the others are labeled as “normal.” Given the positions of the spiked-in mutations, “truth” variant-call format (VCF) files were generated and used as the ground truth against which caller-detected mutations were evaluated. Following this, three simulated tumor-normal paired data sets were generated that are characterized by increasing complexity in regard to mutation types (SNVs, inversions, deletions, duplications, INDELs); increases in the number of SNVs; different variant-allele frequencies; and the incorporation of multiple subclones. We included a fourth data set that was generated the same way as the first three but characterized by even greater complexity regarding the number of SNVs and the level of subclonality (Table 1).

**Table 1:** A summary of the complexity levels of the four synthetic data sets

	Set 1	Set 2	Set 3	Set 4
Mutation types	SNV, structural variation (SV) (deletions, duplications, inversions)	SNV, SV (deletions, duplications, insertions, inversions)	SNV, SV (deletions, duplications, insertions, inversions) & INDEL	SNV, SV (deletions, duplications, inversions) & INDEL
Number of Somatic SNVs	3535	4322	7903	16,268
Cellularity	100%	80%	100%	80%
Subclone variant allele frequencies (VAFs)	N/A	N/A	50%, 33%, 20%	50%, 35% (effectively 30% and 15% due to cellularity)

### Reference files

- human_g1k_v37_decoy [17]
- dbsnp_138.b37 [18]
- b37_cosmic_v54 [19]
- Panel of Normals (PON) from The Broad institute [20]

### Analysis pipeline

The analysis was performed on outputs obtained from five open-source variant-discovery tools accessed on Biowulf, the high-performance computational environment at the National Institutes of Health:

- FreeBayes v1.0, a haplotype-based variant detector for small variations such as single-nucleotide polymorphisms (SNPs) and insertions or deletions (indels) [21]
- VarDict v1.5.1, a variant caller for single and paired samples from BAM files [16, 22]
- MuTect v1.1.7, a caller for detecting somatic point mutations in NGS data derived from cancer genomes [23]
- MuTect2, a variant detector for SNPs and indels that is part of the Genome Analysis Toolkit (GATK) v3.8–0 [24]
- MuSE v1.0rc, a relatively new caller for somatic point mutations in paired tumor-normal samples [25]

FreeBayes, VarDict, and MuTect were run in bcbio-nextgen, v1/0.6, an integrated Python package that provides a fully automated, distributed high-throughput sequence-analysis pipeline and, using a majority-vote approach, integrates the multiple VCF outputs listed above into a single Ensemble call set [26]. MuTect2 and MuSE were run as standalone tools.

To ensure fair, nonbiased comparisons, every tool was used according to its recommended default parameters. The resulting VCF files were compared with the corresponding ground-truth file. Custom scripts were used for data processing, including batch processing and creating a summary of results. Since all five callers detected SNPs, the results are based only on SNP-calling performance. For the integrated approach, bcbio proved easy to configure and run, but it demands extensive resources, including computational power (CPUs) and, often, long stretches of time to run.

To run bcbio-nextgen, a human-readable configuration “YAML Ain’t Markup Language” (YAML) file was generated from a template provided with the tool but customized to a limited extent: calibration and realignment were turned off. A panel of normal VCF files was added to the background parameters. Variants that had passed through the filter and been recorded in the VCF file for each caller were extracted and put into a new VCF file for further analysis.

To run MuTect2, which usually requires a long time to complete, 336-interval Browser Extensible Data (BED) files between sequence-coverage gaps were created so that the tool could be run on paired samples in shorter segments in parallel. Variants that had passed through the filter were merged into a single VCF file for further analysis.

Two steps were necessary to run MuSE. For the first step, the “MuSE call” command was used to take the reference-genome FASTA file and BAM files to complete some pre-filtering steps and finish variant detection by applying the Markov substitution model. For the second step, the “MuSE sump” command was used to turn the output from step 1 into input and to retrieve a bgzip-compressed VCF file from DatabaseSNP. After completing these preliminary steps, MuSE computed tier-based cutoffs (PASS, Tiers 1 to 5) from a sample-specific error model and produced a VCF file. Finally, it extracted the variants that had passed through filters into a new VCF file for further analysis [7].

After variant calling, the VCF files produced by each caller were compared with their corresponding ground-truth files using the evaluator.py script [27], which was provided by the DREAM team referenced above. This script compares the two VCF files to produce true-positive and false-positive rates in relation to the total number of variants detected and the total number of variants in the ground-truth file. It also calculates sensitivity, specificity, and balanced accuracy (sensitivity + specificity)/2) from those numbers.

To demonstrate the extent of the similarities in the results produced by each caller, the open-source visualization tool OmicCircos was employed to reveal overlapping patterns of specific mutations identified by the callers despite the variability of their overall scores (see Fig. 2a and b below) [28]. The bootstrap resampling algorithm was used to evaluate degrees of performance similarity between pairs of callers. N (the observed number of overlaps produced using selected pairs of callers) substitutions were randomly selected and used to resample each data set 10,000 times. Random overlapping numbers were calculated from the N substitutions for each selected pair. In Fig. 2a, the distance between the mean of expected overlapping and observed overlapping values measures the performance similarity between pairs of callers. The shorter the distance on the X axis, the greater the similarity between outputs. Greater similarity is also represented by wider connecting lines between tools in Fig. 2b.

Finally, both the ground-truth and results VCF files were split into 10,000,000 nucleotides to create 316 segments using an in-house perl script. Each segment was compared separately to the corresponding truth file using evaluator.py. To further investigate the effects of G-C content and gene density on caller performance, the G-C content in each segment was obtained using the “bedtools nuc” command, and the gene density of each was obtained using a custom R script.

## Results

### Variant calling

The major advantage of using synthetic data sets is the precise control one can attain of the location and complexity of specific genomic mutations. As shown in Table 1, the four data sets we chose vary in type, in the number of spiked-in mutations, and in the degrees of cellularity and subclonality present, with increasing complexity from set 1 to set 4 resulting from different combinations of these four factors. The variation in data complexity presents different challenges to variant callers and gives us an opportunity to observe how well each caller handles the challenge.

The numbers of true-positive and false-positive SNPs detected by each of the five variant-calling tools plus the Ensemble integrated set (FreeBayes, VarDict, and MuTect) in the four synthetic data sets are presented in Table 2 and compared to the number of SNPs known to exist in the ground-truth file. Differences among the numbers of true positives were small among the callers, but the numbers of false positives varied much more when the tools were used to analyze sets one through three. Both FreeBayes and VarDict produced strikingly more false positives than did the others. Culminating in something of a trade-off, VarDict produced the highest number of true positives but this was undercut since it also produced a high number of false positives. With set 4, the numbers of both true-positive and false-positive calls varied much more than with any other set. Altogether, MuTect2 performed the best among the callers tested, followed by MuSE and MuTect.

**Table 2:** The number of inserted “truth” SNPs in each data set and the numbers detected by each caller. The numbers listed in the first row next to “truth” indicate the number of inserted SNPs in the set

Callers	Set_1 Truth:3535		Set_2 Truth:4322		Set_3 Truth:7903		Set_4 Truth:16268	
	True positive	False positive	True positive	False positive	True positive	False positive	True positive	False positive
FreeBayes	3379	6212	4150	6533	6989	5558	8762	9521
VarDict	3443	6988	4224	6094	7366	5388	12,598	4456
MuTect	3303	755	4081	1176	7100	1098	11,708	1030
Ensemble	3202	199	4085	278	7031	135	10,666	450
MuTect2	3334	384	4087	966	7131	817	11,876	535
MuSE	3447	583	4197	432	6952	299	8941	1353

The Ensemble approach yielded results characterized by higher specificity and balanced accuracy and fewer false positives than did any of the five tools used alone, a not wholly unexpected outcome. Bcbio proved easy to configure and run, but it demands extensive resources, including computational power (CPUs) and, often, long stretches of run time. Further, its intermediate files are frequently quite large, placing extra demands on data acquisition, processing, and storage. In this study, running bcbio produced about one terabyte of intermediate files for each pair of samples.

**Fig. 1:**
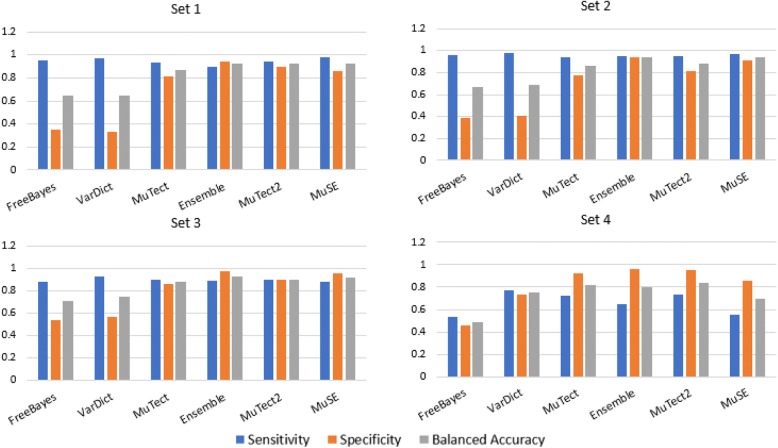
Caller performance comparison in detecting the number of SNPs in each data set is compared to the corresponding numbers in the ground-truth file

The actual numbers determined for each tool and Ensemble provide the basis for the graphic representation of the number of called mutations for which sensitivity (true positives), specificity (false positives), and balanced accuracy (the mean of sensitivity and specificity) scores are shown in Fig. 1.

The graph reflects the diminishing accuracy of caller performance when the tools were exposed to increasing levels of data complexity. Every caller and Ensemble produced their most problematic results when run on data set 4, the most complex set of the four. Regarding the three less-complex sets, sensitivity scores were similar among the tools, but it should be noted again that specificity scores were markedly lower for FreeBayes and VarDict. In consequence, their overall balanced-accuracy scores suffered in comparison to those of the other tools. At the opposite end of the spectrum, Ensemble produced the best scores in most cases, providing support for the observation that integrating calls produced by multiple callers tends to lead to better results.

### Similarity among outputs

**Fig. 2:**
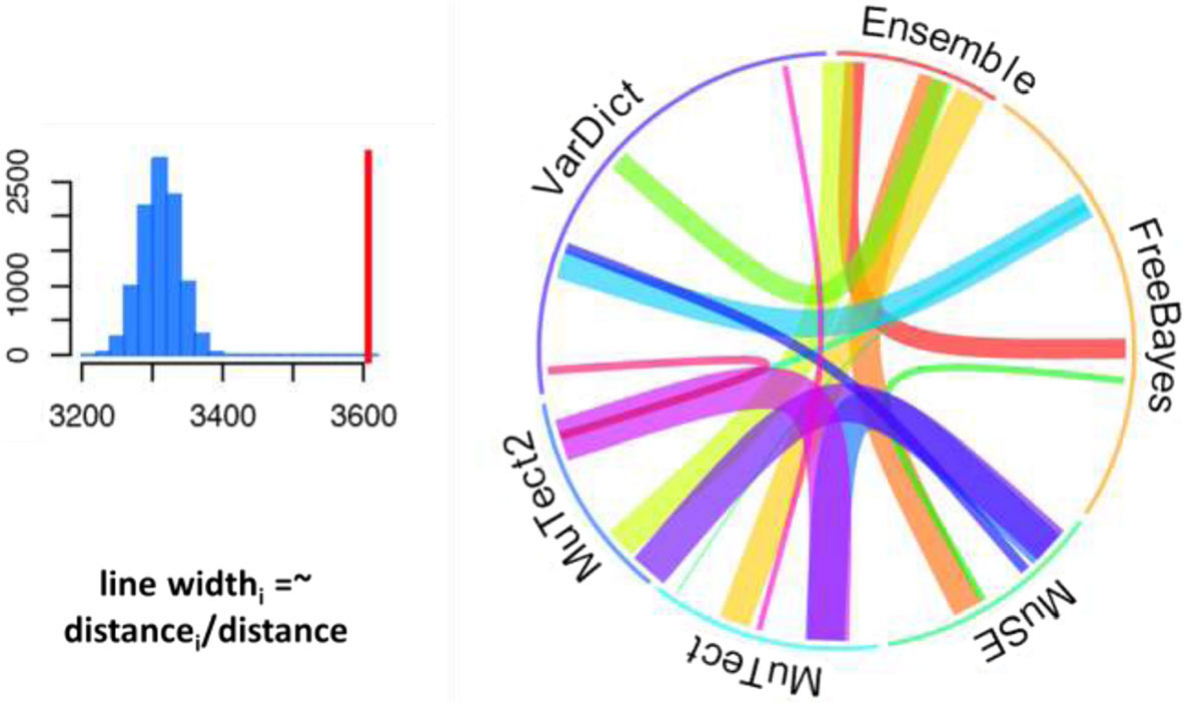
In the resampling process, N substitutions were randomly selected and used to resample each data set 10,000 times. Here, N is the observed overlap number between methods. The random overlap number was calculated from the N substitutions for each selection

We used the bootstrap-resampling algorithm to establish our comparative model and evaluate the degree of performance similarity among callers, as shown in Fig. 2.

In Fig. 2a. The distance between the mean of expected overlapping and observed overlapping values (red bar in 2a) provides a measure of the performance similarity between two callers. The shorter the distance, the greater the similarity between two callers. The similarity between callers is represented by the varying widths of the connecting lines in Fig. 2b, the wider the line, the more similar between the two connected callers.

**Fig. 3:**
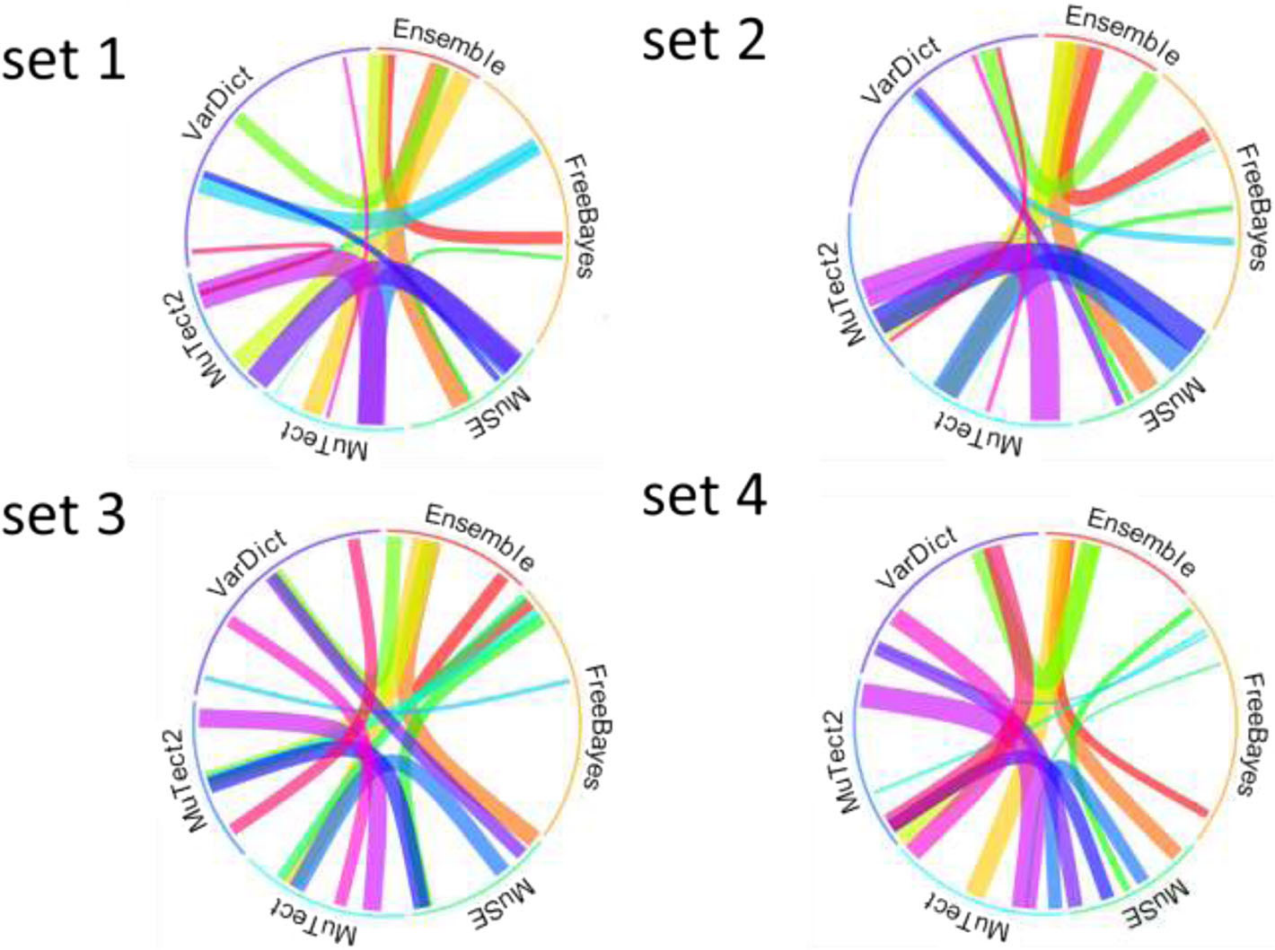
Similarities between callers are represented by the width of the connecting lines

The bootstrap-resampling algorithm was applied to the four data sets, as shown in Fig. 3. Each of the four synthetic data sets is represented by a circular plot ranging from the simplest (1) to the most complex (4). The similarities among Mutect, Mutect2, and MuSE in all four data sets are much higher than with VarDict or FreeBayes. These three callers proved to be our top three performers. In contrast, VarDict and FreeBayes performed similarly only with set 1, the least complex set of the four.

As depicted in Table 3, which represents the pairwise comparison of the five callers and ground truth, the results from data set 1 (a) and data set 4 (b) are presented here. In each row, the number indicates the fraction of SNPs also detected by the caller in the corresponding column. For example, VarDict detected 39.7% of the SNPs identified by FreeBayes given in Row 2 of Table 3a. Among the five callers, FreeBayes and VarDict detected higher numbers of SNVs and exhibited less agreement with the other callers, whereas Mutect, Mutect2 and MuSE detected lower numbers of SNVs and had better agreement with the other callers. This is especially obvious in set 1, where < 40% of SNVs detected by the two callers could be detected by the other callers. Additionally, for set 1 the agreement with truth was better than the agreement between callers, except for Ensemble, which uses a majority-vote post-call methodology. Ensemble has the highest level of agreement with the other callers in regard to sets 1 and 4. The background color reflects the level of agreement between caller pairs: higher-intensity shades of blue reflect higher degrees of agreement.

**Table 3:**
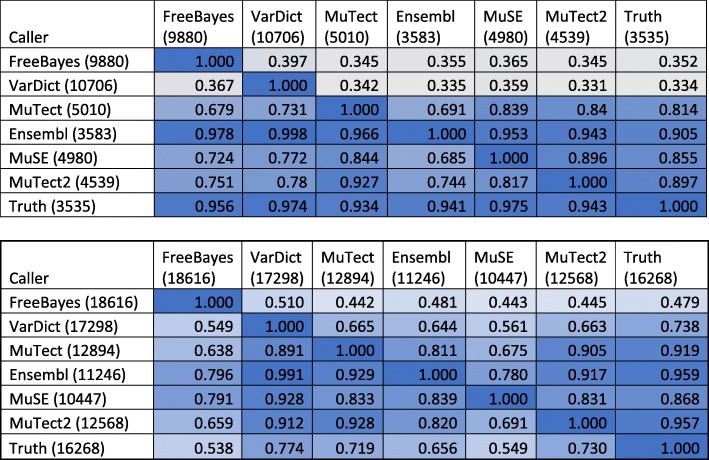
Comparison of outcomes using data sets 1 (3a) and 4 (3b). The background color reflects the degree of agreement between pairs of callers, with greater intensities indicating higher degrees of agreement

### Correlations with sequence structure

To determine whether there might be any relationship between SNPs and G-C content or gene density, we split the genome into 10,000,000 base-pair fragments that yielded a total of 316 segments. SNPs detected in each segment were compared with SNPs in the corresponding segment in the truth file.

**Fig. 4:**
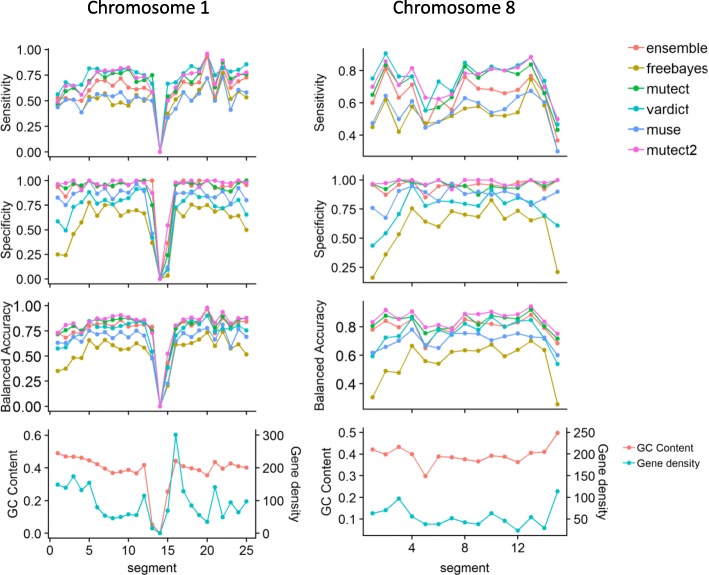
Example plots showing caller performance comparisons of the sequence of segments for chromosomes 1 and 8. SNPs detected in each segment were compared with SNPs in the corresponding segment of the truth file

Sensitivity, specificity, and balanced accuracy were calculated to determine whether there were localized mutational changes with respect to G-C content or gene density. We used segments from chromosomes I and 8 from data set 4 as representative examples in Fig. 4.

**Fig. 5:**
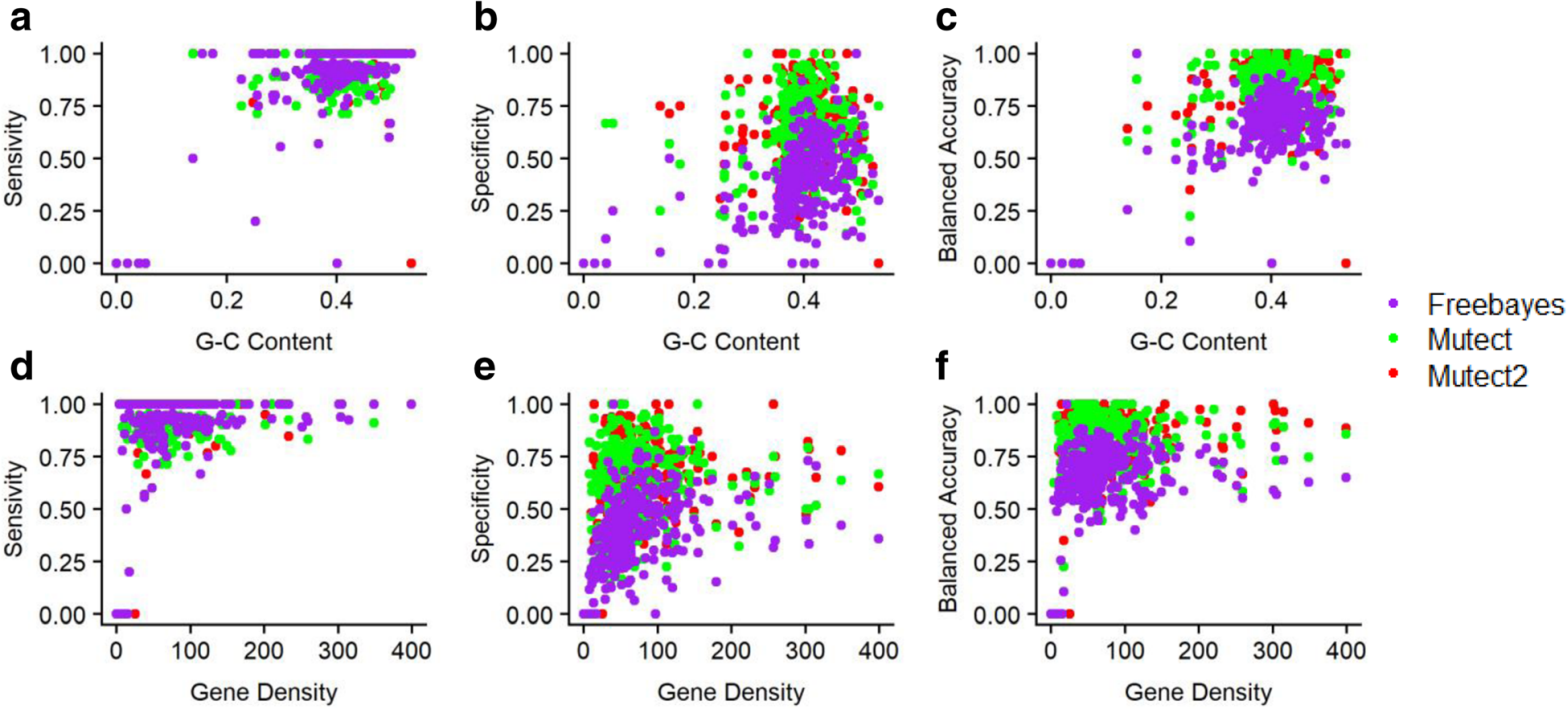
Correlations between sensitivity, specificity and balanced-accuracy scores of three callers to G-C content (**a**, **b**, **c**), and gene density (**d**, **e**, **f**) in set one

Although performance varied among the callers we used and the segments we analyzed, no significant relationships between levels of performance and changing G-C content (a marker of intergenic homopolymeric sequences) or gene density (a measure of the number of genes per the number of base pairs) became apparent. The results from MuTect2, MuTect, and FreeBayes presented in Fig. 5 are representative of the patterns produced by all the callers.

No significant differences were found for correlations between sensitivity, specificity and balanced accuracy with G-C content. Nor was any correlation observed between these factors and gene density. Most segments had a G-C content of about 40%, and gene density was below 100. We did not find any relationship between either G-C content or gene density and detected mutations. This result is not surprising since we used synthetic data in which mutations were inserted randomly. This question merits further investigation to determine whether such a relation exists in real data.

**Fig. 6:**
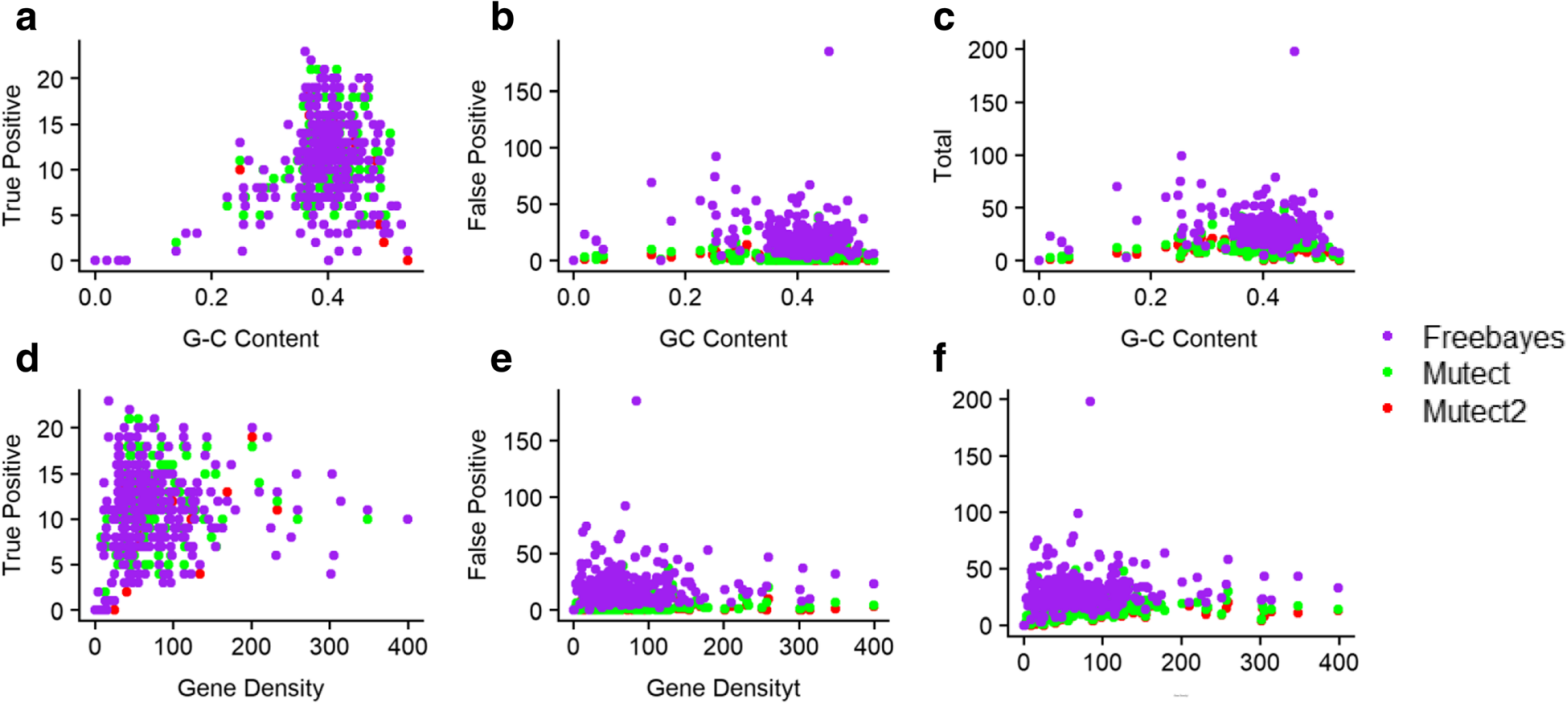
Correlations between true positives, false positives and the total number of SNPs to G-C content (**a**, **b**, **c**) and gene density (**d**, **e**, **f**) in set one

Similarly, to the correlations between sensitivity and balanced-accuracy scores with G-C content in Figs. 5 and 6 represents the correlation between number of true positive, false positive and total number of SNPS to G-C content and gene density. Again, no statistically significant correlations between the number of SNPs detected and gene density became apparent.

## Discussion

We used four synthetic dream-challenge data sets [16] annotated with ground truth for this study. By using synthetic data sets, we had better control of the number and location of genomic mutations as well as better control of data complexity. By including known mutation sites, we resolved the difficulty of lacking ground truth against which to compare mutation callers. Using known sites also allowed us to overcome certain disadvantages inherent in commonly used manual validation procedures [5–7, 29], including time, cost, and most significantly, lack of comprehensiveness. Of particular importance, the inclusion of ground truth enabled us to more accurately estimate true and false negatives.

MuTect2 and MuTect performed comparably. The performance of MuSE was in line with that of MuTect and MuTect2. MuTect2, MuTect, and MuSE proved to be the top three performers in this study. MuSE is a relatively new SNP-detection tool that has not yet been extensively evaluated. We found that it runs relatively fast, especially when running chromosome after chromosome or segment by segment. The performance of FreeBayes was more ambiguous. The caller achieved better sensitivity scores than did MuTect or MutTect2. But this was compromised by much lower specificity scores, and thus, a lower balanced-accuracy score (c.f.).

In general, researchers have found that MuTect and MuTect2 are among the best tools for detecting true positives and controlling for false positives [3, 9]. Although FreeBayes and VarDict did not perform very well in this study, especially in controlling false positives, they may yield better results in other studies [12]. There are different sequencing properties at individual variant sites that challenge a given caller’s ability to detect mutations accurately, including read depth, read quality, strand bias, and varying allele fractions, among others. In this study, the data sets we used vary in cellularity and variant allele fractions. The results show that all callers performed comparably well in detecting true positives at low levels of complexity. The major difference in performance is the ability to minimize false positives, which Mutect2, Mutect and MuSE handled much better than did FreeBayes and VarDict. The increase in data complexity made it harder for all callers to detect true positives, but it had far smaller effects on controlling false positives. Ensemble returned better results than any single caller used alone in most cases, indicating that it takes advantage of every caller’s strength in true-positive detection and false-positive control (see Tables 2 and 3) [6]. This also suggests that there is no “one-size-fits-all” solution for variant calling and therefore provides an additional incentive for using more than one caller and integrating results to improve performance. Although it was not our intention in this study to investigate why different variant callers perform differently on different data sets, we are planning detailed investigations to identify possible contributing factors.

It is thought that higher G-C content regions tend to have higher relative gene density scores than do regions of lower G-C content [30]. But previous studies of the relationship between G-C content and gene expression have shown only a very weak correlation [31]. In this study, we used segmentation analysis attempt to discover whether any correlations between SNP occurrence and G-C content or gene density exist. We found no significant correlations between the two. Our use of synthetic data in this study and the random insertion of SNPs into the genome might account for this result. To resolve this question, we believe it would be worthwhile to carry out a similar analysis using real data sets with known mutation sites. Another possible way to uncover such correlations would be to improve the statistical algorithm we used or to develop new algorithms specifically developed for this kind of analysis. It is also possible that the method we adopted of arbitrarily creating large genomic segments diluted certain small or otherwise subtle SNVs differences between “normal” and “tumor” data sets. Instead of randomly subdividing a genome, one could, for example, perform segmentation based on functional or structural context.

After our initial submission of this manuscript and during its review, we learned that the Global Alliance for Genomics and Health (GA4GH) Benchmarking Team has put together some reference materials and tools for benchmarking germline variant calls [32]. They developed a large set of bed files based on different genomic contexts, including G-C content, coding regions, different types of tandem repeats, and difficult-to-map regions. We plan to examine their approach and may adopt it for somatic variant calls.

In this study, we pursued an unconventional approach to analyzing and comparing the performance of five variant callers not only globally by surveying the entire genome as has traditionally been done, but also by splitting the genome into 316 segments to efficiently search for correlations between localized mutation variations and G-C content or gene density. To our knowledge, this study is among the first, if not the first, to perform whole-genome data analysis on segmented sequences. Sequence segmentation offers distinct advantages, including increased analytical resolution, of which we have taken advantage in this study. Although we did not uncover any major findings in that respect, our approach represents a new way to analyze NGS data. We hope it will inspire the scientific community to pursue this methodological approach further, not only for SNPs but also for other types of mutation, including copy-number variation or shifting methylation patterns using real data sets coupled with ground truth. We also evaluated the performance of MuSE, a relatively new caller, in detecting SNPs. In this study, we found the tool easy to install and run. It runs quickly, and its performance is comparable to some of the best callers such as MuTect2. Finally, we confirmed the finding by others that combining multiple callers can yield better results than does using a single tool in isolation.

## Conclusion

It is incumbent upon the scientific community to reach consensus regarding standards for evaluating the accuracy of new analytical technologies, such as variant callers, used to parse the massive amounts of NGS data we have generated and are transforming into a community resource. The value of NGS data is wholly dependent not only upon accurate analysis and identification of mutations, but also upon valid methods of interpretation and the translation of the resulting data-driven insights into the clinic. To achieve this, we must develop, explore, and compare alternative evaluation methods such as that described in this paper in order to formulate standards of the best-possible quality.
